# A Predictive Approach towards Using PC-SAFT for Modeling the Properties of Shale Oil

**DOI:** 10.3390/ma15124221

**Published:** 2022-06-14

**Authors:** Parsa Mozaffari, Zachariah Steven Baird, Oliver Järvik

**Affiliations:** Department of Energy Technology, School of Engineering, Tallinn University of Technology, Ehitajate tee 5, 19086 Tallinn, Estonia; zachariah.baird@taltech.ee (Z.S.B.); oliver.jarvik@taltech.ee (O.J.)

**Keywords:** Kukersite oil shale, pyrolysis oil, shale oil gasoline fractions, PC-SAFT prediction model

## Abstract

Equations of state are powerful tools for modeling thermophysical properties; however, so far, these have not been developed for shale oil due to a lack of experimental data. Recently, new experimental data were published on the properties of Kukersite shale oil, and here we present a method for modeling the properties of the gasoline fraction of shale oil using the PC-SAFT equation of state. First, using measured property data, correlations were developed to estimate the composition of narrow-boiling-range Kukersite shale gasoline samples based on the boiling point and density. These correlations, along with several PC-SAFT equations of the states of various classes of compounds, were used to predict the PC-SAFT parameters of aromatic compounds present in unconventional oil-containing oxygen compounds with average boiling points up to 180 °C. Developed PC-SAFT equations of state were applied to calculate the temperature-dependent properties (vapor pressure and density) of shale gasoline. The root mean square percentage error of the residuals was 13.2%. The average absolute relative deviation percentages for all vapor pressure and density data were 16.9 and 1.6%, respectively. The utility of this model was shown by predicting the vapor pressure of various portions of the shale gasoline. The validity of this model could be assessed for oil fractions from different deposits. However, the procedure used here to model shale oil gasoline could also be used as an example to derive and develop similar models for oil samples with different origins.

## 1. Introduction

Models to predict the thermodynamic and transport properties of compounds are of interest to many chemical, oil, and related industries. Models to estimate the phase behavior of fluids in the system are used to design chemical processes and equipment, improve separation processes and product quality, and assess the environmental risks that are inevitably associated with these processes. Therefore, the demand for the use of equations and models applicable to complex mixtures of hydrocarbons has increased considerably [1].

While several predictive correlations for thermodynamic properties of complex mixtures such as oils have been suggested, these models were mainly developed for oils containing small concentrations of heteroatoms with aliphatic and aromatic structures. Therefore, these correlations are not particularly applicable to oils with different structures and compositions. The composition of unconventional oils is different from that of petroleum and varies depending on the source of the oil. As a result, the properties are also different. Shale oil is one such unconventional oil. It is produced by thermally processing organic-rich rocks (oil shale). The conversion technique used for oil shale has been known for a century, and is viewed as the most optimal and efficient thermochemical process to convert shale rock into oil. The advantages of this process have also been extended further for conversion of biomass into biofuel both economically and environmentally [2,3].

Although some basic property prediction correlations developed for petroleum could also be used for shale oil [4], in general, proposed correlations and models developed for petroleum do not essentially lead to accurate results for shale oil [5,6,7,8]. Therefore, these models are usually not suitable for shale oil, and more specifically for Kukersite shale oil, due to differences in composition [9]. Information about the properties of shale oil is limited, and thus, from an engineering point of view, developing such prediction models would be beneficial.

Oils are generally complex mixtures with a large number of different compounds. It is not currently feasible to identify all the compounds and their concentrations in the oil; therefore, simplification for modeling is required. This is generally accomplished by lumping compounds together into groups or classes, which are termed pseudocomponents. Once the pseudocomponent is defined, the properties of all the compounds in the pseudocomponent are described using the average properties of the whole group. The oil as a whole is then modeled as a mixture of these pseudocomponents [10].

One of the simplest methods to define pseudocomponents is to split oil into fractions with narrower boiling points, often through distillation, and measure or estimate the properties of those fractions. These methods are sometimes called bulk property methods, and they do not require any information about the composition of the oil. Because it is a labor-intensive process to measure a full set of properties for many fractions, correlations have been developed for petroleum to estimate a variety of properties from a smaller set of experimental data that are commonly measured for an oil. Generally, only the distillation curve and a second property, such as the density, viscosity, or refractive index, need to be measured to be able to model an oil using these methods [10,11].

A more complex method is to analyze the composition of the oil and then use the data to define the pseudocomponents based on the molecular structure. This is commonly performed by splitting the oil into classes of molecules, for instance, using PNA (paraffins, naphthalenes, aromatics) or SARA (saturates, aromatics, resins, asphaltenes) analysis. These types of the composition analyses refer to characterization methods used to quantitatively determine the amount of each class of compound in an oil. These classes are then further divided by the size of the molecules, i.e., average molar mass or the average number of carbon atoms [10]. The properties of these pseudocomponents can then be estimated based on existing data for pure compounds with the same type of structure. For instance, the properties of paraffin pseudocomponents can be calculated from the properties of pure n-alkanes. For petroleum, there are even correlations that allow the composition of oil to be calculated based simply on the measured properties of an oil and its fractions [10]. Modern analytical techniques, such as gas chromatography and mass spectroscopy, can provide even more detailed data, allowing the pseudocomponents to be defined closer to the level of individual compounds [12]. However, these analytical techniques are expensive and time-consuming, so in industry, the simpler characterization schemes are usually used [11].

If possible, the goal is often to model the pseudocomponents and the oil as a whole using an equation of state. Equations of state allow many of the properties of a mixture to be modeled over a wide range of temperatures and pressures. Correlations for predicting the equation of state parameters for petroleum pseudocomponents have been developed with this goal in mind [10,13]. In the past, cubic equations of state have often been used. However, cubic equations of state require values for the critical properties of a pseudocomponent, and these properties can be difficult to measure or estimate accurately [10]. In the last two decades, it has become more common to use equations of state based on statistical associating fluid theory (SAFT), especially perturbed-chain statistical associating fluid theory (PC-SAFT), for modeling oils, including bio-oils [1,13,14,15,16,17,18]. Moreover, several studies indicate the importance and applicability of these equations in the energy industry [19,20,21].

SAFT models do not require critical property values and they can more accurately calculate the liquid density of a system, which means that density data can also be used in fitting PC-SAFT parameters [1]. Indeed, some systems have been modeled using only density data for parameter fitting [22].

Modeling shale oil has received relatively little attention. As previously stated, because shale oil has a much different composition than petroleum, the validity of correlations and models developed for petroleum is predominantly questionable for shale oil. Based on our literature survey, the only correlations that attempt to provide some sort of systematic modeling framework for shale oil were published in 1930 by Kogerman and Kõll [23], in 1934 by Luts [24], and in 1951 by Kollerov [25]. All three of these publications rely in large part on the experimental data from Kogerman and Kõll [23], which was from only a single distillation of oil from an experimental generator retort. These studies focused mainly on shale oil from Estonian Kukersite oil shale, and shale oils from other deposits have received even less attention.

One of the main obstacles to developing correlations for shale oil has been the lack of data [26]. The new data that have been measured now provide an opportunity to perform this modeling work [27]. Here, we provide correlations that allow gasoline fractions of Kukersite shale oil to be defined in terms of pseudocomponents, and then we present equations for calculating the PC-SAFT parameters of these pseudocomponents. Note that, based on the type of oil shale and the pyrolysis method used, the properties of shale oils can change greatly [28,29,30,31,32]. Therefore, the models developed for Kukersite gasoline shale oil probably cannot be directly used for modeling shale oils from other deposits. However, the approach developed for this purpose could be generally applied to model other shale oils.

## 2. Modeling Methods

### 2.1. Industrial Samples

Several wide Kukersite shale oil gasoline fractions with a boiling range from about 40 to about 200 °C were obtained from Eesti Energia’s Oil Plant (Narva, Estonia). The technology developed for processing oil shale in this plant was described by Neshumayev et al. [33]. These wide fractions were then separated into narrow-boiling-range fractions using different distillation methods. The sample preparation and distillation methods were previously described by Järvik et al. [34]. These fractions were received from the plant at different times in the hopes of capturing the natural range of variation in the composition of shale gasoline. Over this two-year period, 6 different distillations were performed to fractionate the gasoline samples received. These differences in composition and distillation method should help ensure that the model works for a variety of Kukersite gasoline samples.

### 2.2. Compositional Analysis

Generally, the properties of oil fractions depend on the chemical composition of these fractions. Obtaining a relationship between chemical composition and the properties of gasoline fractions could allow the composition of a sample to be predicted from basic physical properties. This is important because PC-SAFT models generally require information about the composition.

Several investigations were previously carried out on the composition and characteristics of Kukersite shale oil [35,36,37,38,39,40]. In these works, the chemical composition of Kukersite shale gasoline was provided for different retorts. Having considered these data, the compositions (mass%) of gasoline fractions used in this work were estimated, mainly based on the detailed data that are partially available in the study published by Gubergrits et al. [34,40]. In this study, the same technology (the Galoter process) was used to obtain shale oil. In Estonia, this technology was developed later than other processes and it is currently the main technology used to produce shale oil.

For the gasoline fractions studied in this work, different properties such as the hydrogen–carbon ratio, infrared spectra, and hydroxyl group content were measured. The behavior of these properties was previously discussed [34]. Almost all the measurements were repeated at least once and if large difference was observed, then additional measurements were carried out for better reliability. Based on these properties, reasonable assumptions about the composition were made so that the changes in the main classes of compounds with average boiling points, up to 180 °C, were carefully defined.

In addition to aromatics, olefins, and paraffins, the rest of the compounds were assumed to be oxygen-containing compounds. Based on the experimental data for gasoline fractions distilled below 180 °C, the concentration of phenolic compounds is quite low; therefore, phenolic compounds were disregarded for further analysis and modeling. Therefore, for developing the model, four different classes of compounds (olefin, paraffin, aromatic, and oxygen-containing compounds) were considered in total. The oxygen-containing compounds are mostly ketones, aldehydes, and ethers [41]. The change in composition of each class of compound was estimated for different average boiling temperatures. From 40 °C to 180 °C, the amount of each class of compound (i.e., olefin, paraffin, and aromatic and neutral oxygen) was carefully estimated so that these changes were consistent with the FTIR and elemental composition analysis of the studied gasoline fractions. For estimation of the amount of each class of compound, chemical group composition data provided by Luik [42] was also considered. It should be noted that the compositions provided in this work are just estimates and the exact composition is unknown because such detailed data have not been measured before. Although the objective of the present study was to develop a model for Kukersite shale oil gasoline with average boiling points up to 180 °C, the composition was estimated up to 500 °C to facilitate future studies where the modeling of all Kukersite shale oil fractions is of interest. This helps to deliver a systematic and coherent path to extend the study. However, in this study, we focused solely on shale gasoline fraction because additional variables and adjustments are required to model the phenols in the heavier portion of the oil. In other words, the analysis performed in this study is the first step in a multistep process.

It was noticed that numerous fractions have similar boiling points, but differ in other properties, such as density. Because oil samples with the same boiling range can still have different compositions, the effect of density along with boiling point was also included as a second parameter to better model the variations of composition that occur in shale oil. Therefore, the composition of each class of compound was estimated such that these variations were also taken into account. For instance, having considered fractions with similar boiling points, fractions with lower densities are expected to have more olefins and paraffins and fewer aromatic compounds in their composition. To incorporate density into the correlations for composition, first, the densities of all fractions were plotted versus their average boiling point, and this trend was used to calculate the average density at a given boiling point. Compositions were also estimated for fractions with similar boiling points and higher or lower densities. The result was a dataset of fractions with different boiling points and densities along with estimates of their compositions.

The estimated mass percent of each class of compound with respect to average boiling point and density were then fitted using the differential evolution optimizer in the Scipy package for Python [43,44]. All experimental data are available in [27], and the basic correlation considered to fit the variables is as follows:

(1) $${X=C}_{0}T_{b}^{2}{+C}_{1}T_{b}{+C}_{2}{+C}_{3}\rho^{2}{+C}_{4}\rho {+C}_{5}T_{b}\;\rho \;$$

In Equation (1), *X* is the mass percent of the class of compound, *T_b_* is the average boiling point (°C), ρ is the density (kg m^−3^), and *C*_0_–*C*_5_ are constants obtained from the fitted data. The concentration for neutral oxygen compounds was calculated by difference. Therefore, separate coefficients were not obtained for neutral oxygen compounds.

### 2.3. Shale Oil Modeling

The shale oil gasoline was modeled using the PC-SAFT equation of state. The PC-SAFT equation of state was thoroughly described by Gross and Sadowsky [1]. This equation is used to predict the thermodynamic behavior of pure and multicomponent systems. For the PC-SAFT equation, the main parameters characterizing a fluid are the segment number (m), segment diameter (σ), and segment energy (ε/k). For neutral oxygen compounds, we also included a polar term, which depends on the dipole moment. The dipole moment for many ketones and aldehydes is 2.7 [45], so this value was used in the model.

These parameters for the aromatic class of compounds were determined by fitting the PC-SAFT equation of state to the measured physical property data, i.e., liquid density, and the average normal boiling point of the fractions. For data analysis of experimental data and model development, Python (Version 3.9) was used.

For the purpose of developing a model, it was shown by Gross and Sadowsky [1] that Equations (2)–(4) are suitable for correlating parameters for pure compounds with varying molar masses.

The relation for segment diameter (*σ_i_*) as a function of the molecular weight (*M_i_*) is as follows:

(2) $$\sigma_{i}{=q}_{01\;}{+q}_{11}\frac{M_{i}{\;-\;M}_{{CH}_{4}}}{M_{i}}{+q}_{21}\frac{M_{i}{\;-\;M}_{{CH}_{4}}}{M_{i}}\frac{M_{i}{\;-\;2M}_{{CH}_{4}}}{M_{i}}\;$$

For the chain length to molecular weight ratio, the proposed relation is:

(3) $$\frac{m_{i}}{M_{i}}{=q}_{02}{+q}_{12}\frac{M_{i}{\;-\;M}_{{CH}_{4}}}{M_{i}}{+q}_{22}\frac{M_{i}{\;-\;M}_{{CH}_{4}}}{M_{i}}\frac{M_{i}{\;-\;2M}_{{CH}_{4}}}{M_{i}}\;$$

In addition, the relationship for the dispersion energy parameter is given as:

(4) $$\frac{\varepsilon_{i}}{k}{=q}_{03}{+q}_{13}\frac{M_{i}{\;-\;M}_{{CH}_{4}}}{M_{i}}{+q}_{23}\frac{M_{i}{\;-\;M}_{{CH}_{4}}}{M_{i}}\frac{M_{i}{\;-\;2M}_{{CH}_{4}}}{M_{i}}\;$$

Here, $M_{{CH}_{4}}$ is the molecular weight of methane ($M_{{CH}_{4}}$ = 16.043 g mol^−1^) and *q_jk_* are constants to be fitted to pure component parameters (*i* refers to component *i*). For the n-alkane series, these constants were previously published by Gross and Sadowski [1], and these suggested relations were used as a model for paraffins in shale oil. Moreover, Ghosh et al. [46] proposed correlations obtained by fitting the homologous series of 1-alkenes; therefore, these relations were also used for olefin compounds in Kukersite shale gasoline fractions. Correlations for the neutral oxygen compounds (ketones) were obtained from linear regression between PC-SAFT parameters of several pure compounds and their molecular weights. This was implemented to find the line of best fit. Data for neutral oxygen compounds was obtained from the work published by Kleiner and Sadowski [45]. The form of the equation for neutral oxygen compounds and aromatic compounds differed from that of other compounds, for which Equations (2)–(4) were used. Below, the suggested equations for oxygen-containing compounds are shown:

(5) $$f\left(MW\right)=\left\{\begin{array}{c} {m=C}_{6}{\;MW+C}_{7}\\ {\sigma =C}_{8}{\;MW+C}_{9}\\ \frac{\varepsilon }{\;k}{=C}_{10}{\;MW+C}_{11} \end{array}\right.$$

where $C_{6}$ to $C_{11}$ are coefficients obtained from the fit and *MW* is the molecular weight of the pure compounds used (g mol^−1^).

For aromatic compounds, there are numerous correlations suggested in the literature for pure compounds and petroleum cuts. However, existing correlations yielded poor results when tested for these shale gasoline fractions. This could be expected because aromatic compounds in Kukersite shale oil might not be similar to the pure compounds used for literature correlations. Therefore, although an equation form from the literature was used, the coefficients were optimized to give better results for Kukersite shale oil. The following relations (Equations (6)–(8)) for pure component aromatic compounds were taken from the work published by Gonzalez et al. [47]:

(6) $$m_{aromatic}{=q}_{01\;}{MW+q}_{11\;}\;$$

(7) $$s_{aromatic}=\frac{q_{02\;}{\;MW+q}_{12}}{m_{aromatic}}\;\;$$

(8) $$e_{aromatic}{=q}_{03\;}{\log\;(MW)+q}_{13\;}\;$$

**Figure 1:**
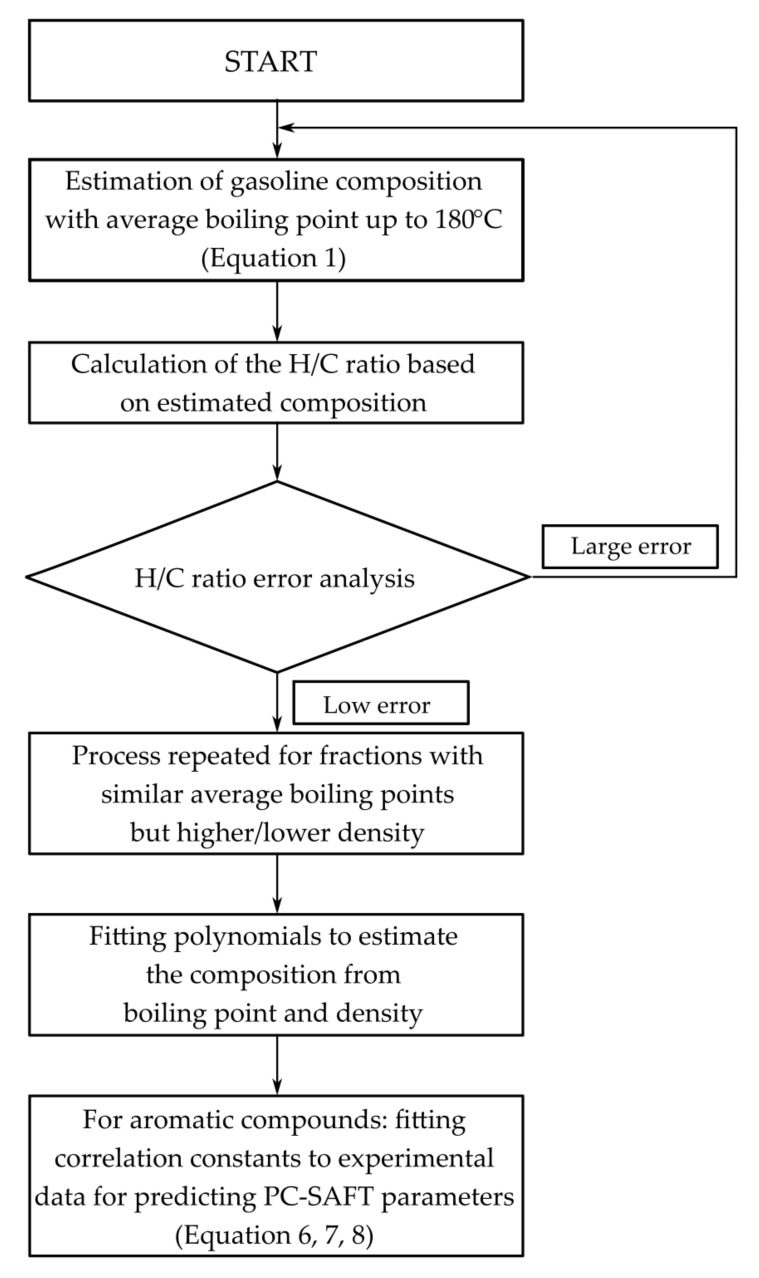
The modeling procedure used in this work for shale oil gasoline fractions.

The coefficients of the correlations for aromatic compounds were fit to experimental data for shale gasoline fractions. The scheme in Figure 1 summarizes the full process to model shale oil gasoline fractions, including the development of correlations for predicting the composition of shale gasoline samples, which were used in developing the PC-SAFT model.

The estimated hydrogen–carbon ratio was compared with the actual hydrogen–carbon ratio and the average absolute deviation was obtained to be 1.6%.

## 3. Results and Discussion

Table 1 shows the coefficients for olefins, paraffins, aromatics, and neutral oxygen compounds. These coefficients were used to predict the composition of narrow-boiling-range fractions. Coefficients *C*_0_–*C*_5_ for Equation (1) were regressed for olefin, paraffin, and aromatic compounds. Then, the content of neutral oxygen compounds was calculated by subtracting the content of other compounds from the total.

Using experimental vapor pressure and density data for gasoline fractions up to 180 °C, correlation constants were optimized for predicting the PC-SAFT parameters of aromatic compounds in Kukersite shale gasoline. These constants are shown in Table 2 along with the coefficients from the literature for paraffins and olefins. For most of the fractions, densities were measured at different temperatures, while vapor pressure was only used at the normal boiling point.

Coefficients C_6_ to C_11_ for oxygen-containing compounds were found from Equation (5), in which PC-SAFT parameters were linearly fit to the molecular weight of several ketones and aldehydes. These coefficients are given in Table 3.

The root mean square percent error (RMSE) for all compounds was found to be 13.2%. This is a reasonable accuracy for a model for oil samples, especially since the wide fractions were taken at different times from the oil plant; therefore, the properties of these fractions varied. Additionally, different distillation types were used to obtain narrow boiling samples from these wide fractions. These differences ensure that a wide variety of samples and properties were used, and thus they ensure that the model developed for shale gasoline fractions could be used for a broader range of samples. The error for the three-parameter equation of state can be considered reasonable for these types of oils, considering the complexity of these mixtures and the lack of data on the detailed composition of shale oils. If the results of the prediction model for different properties are considered separately, then the RMSE for density was much lower than that of vapor pressure.

**Figure 2:**
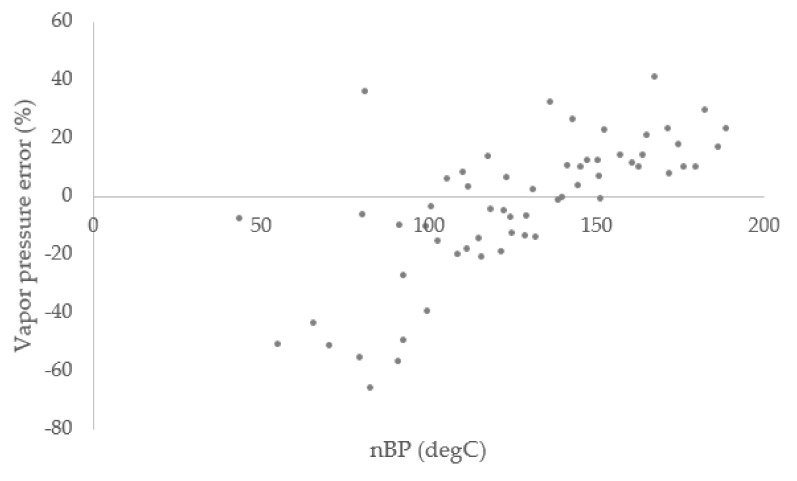
Percent error of the vapor pressure calculated using PC-SAFT for all gasoline fractions analyzed in this work.

**Figure 3:**
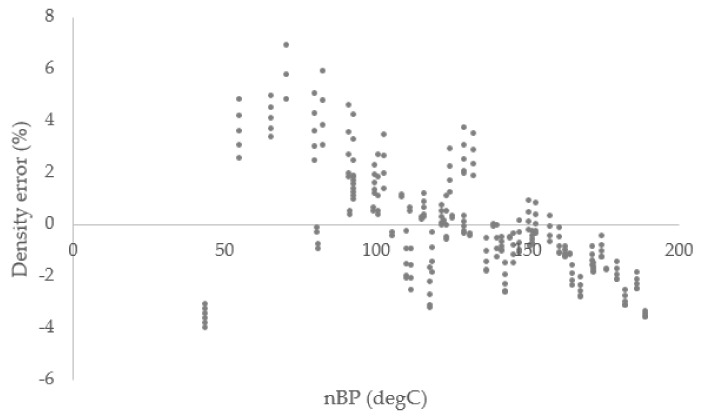
Percent error of the calculated density values.

Figure 2 and Figure 3 illustrates the error percent for the vapor pressure and density of all gasoline fractions calculated using the PC-SAFT equation of state. In these figures, the *x*-axis indicates the normal boiling points (nBP) of the gasoline fractions. The smallest errors are mostly for calculated density values. While many errors for individual data points are below 10%, there are several data points that show higher deviations. These outliers comprise 13% of the total data points used for modeling. All these data points with large errors are vapor pressures and for samples with normal boiling points below about 100 °C. For these lower boiling points, one factor contributing to the larger relative errors is that the absolute value of the boiling point is smaller.

**Figure 4:**
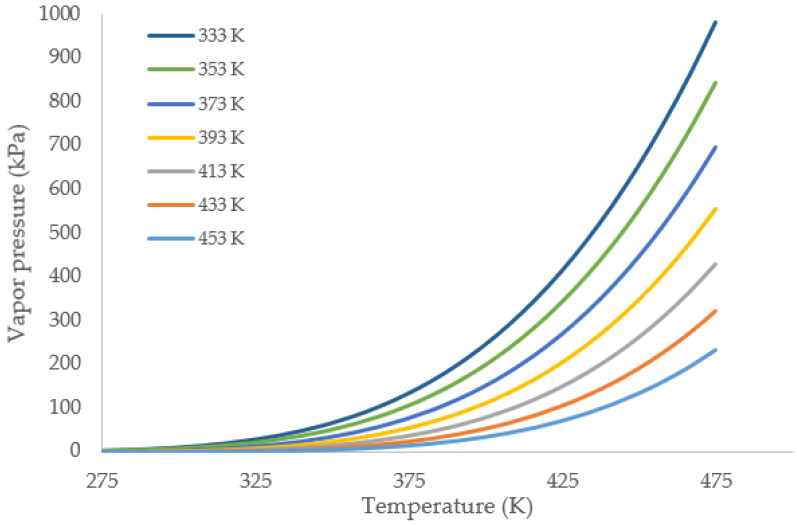
Predicted vapor pressure of gasoline fractions with varying normal boiling points. The values in legends are temperature in Kelvin.

Using the model, the vapor pressures of gasoline fractions with different average boiling points were predicted and the results are shown in Figure 4. The vapor pressure curves were plotted from approximately 60 to 180 °C. Hypothetical fractions with boiling temperatures were plotted in 20 °C increments in order to present the whole range of light distillates.

Overall, larger deviation was seen for lower boiling fractions below 100 °C (Figure 2). The average absolute relative deviation percentage (AARD%) between model values and experimental values was calculated using the below equation:

(9) $$AARD\%=\frac{100}{n}\overset{n}{\underset{i=1}{\sum }}\left(\;\frac{\left|x_{exp}{-\;x}_{calc}\right|}{x_{exp}}\right)$$

In Equation (9), $x_{calc}$ is the calculated property value (vapor pressure or liquid density) using the model, $x_{exp}$ is the measured (experimental) value, and n is total number of data points.

The AARD% for all vapor pressure data was 16.9% and this deviation reduced to 11.6% for fractions with average boing points above 100 °C. Additionally, for density prediction, the average absolute deviation was 1.6%, which indicated considerable reliability of the model.

**Figure 5:**
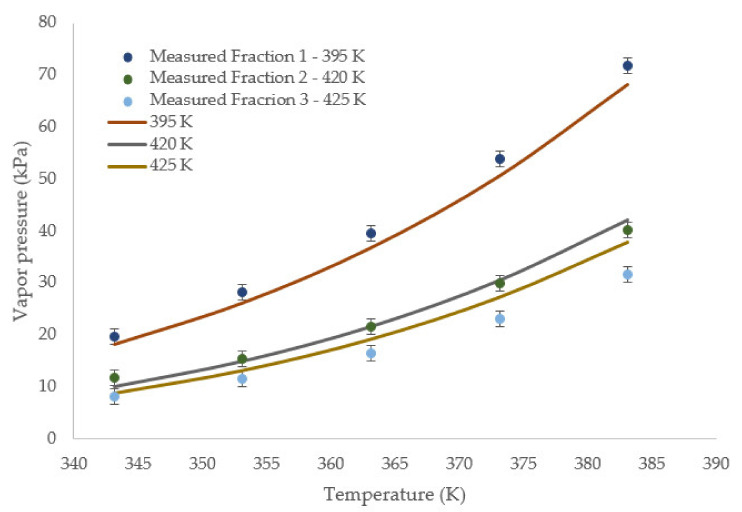
Comparison of measured and calculated vapor pressure of several gasoline fractions. The values in legends are temperature in Kelvin. Error bar shows expanded uncertainty (95% confidence level, k = 2) of measured vapor pressure values.

Furthermore, as for comparison with the model, several gasoline fractions were analyzed, and the vapor pressure curves of these fractions were plotted and compared with calculated curve in Figure 5. Some characteristic properties of these fractions were as follows: fraction 1 (T_b_ = 395 K, ρ = 792.2 kg m^−3^, *MW* = 112.4 g mol^−1^), fraction 2 (T_b_ = 420 K, ρ = 809.6 kg m^−3^, *MW* = 122 g mol^−1^), fraction 3 (T_b_ = 425 K, ρ = 818.0 kg m^−3^, *MW* = 126 g mol^−1^). The vapor pressures of these fractions were obtained and compared from about 343 K to 383 K. The expanded uncertainty of vapor pressure measurements at 95% confidence level (k = 2) was found to be 1.5 kPa. Within the experimental temperature range, the largest AARD% for fraction 1 was seen to be 6.8%. However, corresponding absolute deviation was 3.4 kPa. This could be expected due to the low vapor pressure of this fraction. Of all the experimental values for fraction 1, the largest absolute deviation was seen to be at 3.7 kPa. For fractions 2 and 3, the AARD% were 4.8 and 15.8%, respectively. However, despite the larger AARD% for fraction 3, the average absolute deviation was 3.1 kPa.

In general, the developed model showed favorable results when modeling shale oil gasoline fractions. With this analysis as a basis, modeling could be further extended to shale oil fractions with normal boing points above 180 °C [29,34] in the future.

## 4. Conclusions

In this work, we presented the developed PC-SAFT equation of state model for predicting the 35 gasoline fractions. The model is using normal boiling point and density at 20 °C of a fraction as input parameters. These input parameters were used to estimate the composition of the fraction and consequently to calculate the temperature dependence of vapor pressure and density of shale oil samples. Based on literature data, shale gasoline fractions were assumed to contain four main classes of compounds: olefins, paraffins, aromatics, and oxygen-containing compounds. For estimating the composition, simple polynomial equations were developed using available literature data for the composition of Kukersite shale oil gasoline. Ready-to-use correlations from available literature for olefins and paraffins along with linear equations obtained for oxygen-containing compounds were used to develop respective correlations for aromatic compounds. The resulting model can be used as a property prediction model for shale gasoline samples with normal boiling points below 180 °C.

The suitability of these prediction models for Kukersite shale gasoline was evaluated and the root mean square percent error was 13.2%. Although good results were obtained for Kukersite shale oil, due to the difference in the composition for different shale oils, the applicability of the model could be further assessed once the composition of the main classes of compounds of other shale oils is analyzed.

## Figures and Tables

**Table 1:** Correlation constants for predicting the composition of all classes of compounds present in Kukersite shale gasoline fractions.

Classes of Compounds	C_0_	C_1_	C_2_	C_3_	C_4_	C_5_
Olefins	0.00019606	−0.06643364	219.60530800	0.00016198	−0.33551954	−0.00011084
Paraffins	0.00012930	−0.08110412	173.77635700	0.00012325	−0.27158847	−0.00002634
Aromatics	−0.00015131	0.16682950	−52.31917130	−0.00003139	0.10378634	−0.000046658

**Table 2:** PC-SAFT correlation constants used for aromatics, paraffins, and olefins.

Correlation Constants	Unit	Aromatic	Olefin *	Paraffin **
q_01_	Å	0.0230	3.7146	3.7039
q_11_		0.6411	−0.4797	−0.3226
q_21_			0.8790	0.6907
q_02_	mol g^−1^	0.0823	0.07901	0.06233
q_12_		3.3062	−0.05266	−0.02236
q_22_			−0.00175	−0.01563
q_03_	K	57.7375	121.09	150.03
q_13_		149.9793	133.62	80.68
q_23_			15.648	38.96

* The coefficients were published by Gross and Sadowsky [1]. ** The coefficients were published by Ghosh et al. [46].

**Table 3:** Correlation constants for neutral oxygen compounds in Kukersite shale gasoline.

C_6_	C_7_	C_8_	C_9_	C_10_	C_11_
0.02796	0.90944	0.00244	3.27518	0.05441	242.10097

## Data Availability

The supporting data is available in: https://osf.io/fqjhd/.
